# Autoantibodies in Autoimmune Pancreatitis

**DOI:** 10.1155/2012/940831

**Published:** 2012-07-12

**Authors:** Daniel S. Smyk, Eirini I. Rigopoulou, Andreas L. Koutsoumpas, Stephen Kriese, Andrew K. Burroughs, Dimitrios P. Bogdanos

**Affiliations:** ^1^Institute of Liver Studies, King's College London School of Medicine, King's College Hospital, Denmark Hill, London SE5 9RS, UK; ^2^Department of Medicine, University of Thessaly Medical School, Viopolis, 41110 Larissa, Greece; ^3^The Sheila Sherlock Liver Centre and University Department of Surgery, Royal Free Hospital, London NW3 2QG, UK; ^4^Research Group of Cell Immunotherapy and Molecular Immunodiagnostics, Institute of Biomedical Research & Technology, Centre for Research and Technology-Thessaly (CE.RE.TE.TH), 41222 Larissa, Greece

## Abstract

Autoimmune pancreatitis (AIP) was first used to describe cases of pancreatitis with narrowing of the pancreatic duct, enlargement of the pancreas, hyper-**γ**-globulinaemia, and antinuclear antibody (ANA) positivity serologically. The main differential diagnosis, is pancreatic cancer, which can be ruled out through radiological, serological, and histological investigations. The targets of ANA in patients with autoimmune pancreatitis do not appear to be similar to those found in other rheumatological diseases, as dsDNA, SS-A, and SS-B are not frequently recognized by AIP-related ANA. Other disease-specific autoantibodies, such as, antimitochondrial, antineutrophil cytoplasmic antibodies or diabetes-specific autoantibodies are virtually absent. Further studies have focused on the identification of pancreas-specific autoantigens and reported significant reactivity to lactoferrin, carbonic anhydrase, pancreas secretory trypsin inhibitor, amylase-alpha, heat-shock protein, and plasminogen-binding protein. This paper discusses the findings of these investigations and their relevance to the diagnosis, management, and pathogenesis of autoimmune pancreatitis.

## 1. Introduction

Autoimmune pancreatitis (AIP) is a form of chronic pancreatitis with raised levels of serum IgG4, responsiveness to immunosuppressive therapy, and no apparent underlying cause such as chronic alcoholic pancreatitis [1–8]. Although first described in 1961 as a case of pancreatitis with autoimmune features [9], the term AIP was first used to describe a case involving diffuse enlargement of the pancreas, irregular narrowing of the pancreatic duct and serological markers of hyper-*γ*-globulinaemia, as well as antinuclear antibody (ANA) positivity by indirect immunofluorescence (IIF) [10]. AIP is subclassified in two types: IgG4-related (type 1) and non-IgG4-related (type 2). Type 1 is more prevalent in Asia, whereas type 2 appears to have a higher prevalence in Europe, followed by the USA then Asia [7]. In this paper, AIP will refer to type 1. Patients with AIP are normally responsive to immunosuppressive therapy [1–7, 9–11].

AIP predominantly affects males of middle age [12–14], with the most common presenting symptom being obstructive cholestasis [15, 16]. Laboratory investigations usually reveal hyperbilirubinaemia, raised ALP and transaminases, and occasionally raised carbohydrate antigen 19-9 (CA19-9) [7]. Approximately half of cases have elevated levels of pancreatic enzymes [17]. Elevated IgG4 (>135 mg/dL) is the hallmark of AIP, being elevated in more than 90% of patients [18]. The elevation of IgG4 has been confirmed in several studies [19–21]. The major differential diagnosis is pancreatic cancer, which is usually ruled out through radiological, serological, or histological investigation [22, 23]. Diffuse pancreatic enlargement with a capsule-like rim and narrowed pancreatic duct is suggestive of AIP over cancer, as are delayed enhancement and downstream dilation of the pancreatic duct [24]. Diffuse, solitary or multiple areas of signal hypersensitivity on diffusion-weighted MRI are characteristics of AIP, as opposed to solitary signals in pancreatic cancer [24]. Also, IgG4 levels greater than 280 mg/dl are typically found in AIP as opposed to pancreatic cancer [17], as is IgG4+ immunostaining of the duodenal papilla [25, 26]. 

Although a variety of diagnostic criteria have been proposed [27–29], a firm diagnosis of AIP is based on radiological, serological, and histological findings. Typical radiological features include diffuse pancreatic enlargement with a narrowed pancreatic duct [7, 29]. These features, in the presence of a raised IgG4, are diagnostic of AIP [7, 29]. Features of autoimmune disease may also be present and include the presence of a variety of autoantibodies and/or raised IgG [7, 29]. Immunostaining for IgG4 with ≥10 IgG4^+^ plasma cells per high-power field supports a diagnosis of AIP [30]. A purely histological diagnosis of AIP requires the presence of four features; (1) prominent lymphoplasmacytic infiltration with fibrosis but no neutrophil infiltration, (2) prominent IgG4^+^ plasma cell infiltration, (3) storiform/swirling fibrosis, and (4) obliterative phlebitis [7]. Histopathological diagnosis is particularly important for retrospective reviews of cases without sufficient serological and/or radiological data. It is currently unknown whether findings of a needle biopsy are sufficient for the diagnosis. 

In addition to its role in the diagnosis of AIP, IgG4 also appears to relate to the disease severity and clinical course. Matsubayashi et al. [4] evaluated the clinical course of AIP in relation to IgG4 levels, in a cohort of 27 AIP patients. Elevated IgG4 was present in 74% of the patients, compared to normal IgG4 in 26% [4]. Jaundice was more prevalent in patients with raised IgG4 (80% versus 14.3%), as was weight loss (40% versus 14.3%) [4]. However, abdominal/back pain was reported more in the group with low IgG4 (71.4% versus 50%), as was fatigue (42.9% versus 40%) [4]. Although steroid therapy was successful in both groups, 85.7% of those with raised IgG4 required maintenance therapy compared to 33.3% of those with normal IgG4 [4]. Despite clinical improvement after immunosuppression being observed in other studies [5, 31–34], no consensus has been reached in regards to treatment requirements/alterations in those with higher IgG4 levels compared with those with lower levels. 

Although pancreatic involvement is the major feature of AIP, lesions may also be present in various other organs [3, 35, 36], including (but not limited to) the lachrymal and salivary glands [3], lungs [37], retroperitoneum [19], and prostate [1, 38]. Sclerosing cholangitis is the most common extrapancreatic lesion and is practically associated with the IgG4 form of the disease (IgG4 sclerosing cholangitis) [24]. These lesions are histologically similar to those found in the pancreas [24], clearly indicating that AIP is a pancreatic manifestation of a systemic IgG4 disease [24, 39, 40]. 

A number of autoantibodies have been detected in AIP patients. The presence of autoantibodies supports the prevailing notion that AIP is an immune-mediated and/or autoimmune disease. This paper describes the current data surrounding the presence and role of autoantibodies in AIP. 

## 2. Pathogenesis of AIP: The Role of ****Autoantibodies

Several genetic susceptibility factors for AIP have been identified [41–44], and the disease is now believed to be autoimmune. The autoimmune hypothesis surrounding IgG4-related disease has initiated a series of studies investigating the specificity of autoantibody responses in patients with AIP leading to the identification of several autoantigens/autoantibodies. Several autoantibodies have been found in AIP patients (see Table 1 for a list of the major autoantibodies found), some more prevalent than others. These autoantibodies can be subdivided into two broad categories consisting of nonorgan and organ-specific autoantibodies. Organ-specific autoantibodies have attracted special attention because of their potential pathogenetic relevance to the initiation of the disease, but most of them are not highly prevalent in AIP or are seen in low titers. The broad variety of antibodies include antilactoferrin (anti-LF) [45, 46], anticarbonic anhydrase II (anti-CA-IIAb) [47, 48], anticarbonic anhydrase IV (anti-CA-IVAb) [49], antipancreas secretory trypsin inhibitor (anti-PSTI) [50], antitrypsinogens [51], antiamylase alpha [52], anti-heat shock protein 10 (anti-HSP10) [53], and antiplasminogen-binding protein peptide (anti-PBP) [54]. None of these autoantibodies have been considered disease specific, and this may be one of the distinctive features of AIP compared to other gastrointestinal and liver autoimmune diseases characterized by disease specific autoantibodies, including antimitochondrial antibodies (AMA) in patients with primary biliary cirrhosis (PBC) or anti-liver kidney microsomal-1 (anti-LKM1) antibodies in type 2 autoimmune hepatitis. Up to 40% of patients has detectable rheumatoid factor (RF) and ANA [5, 6]. Yamamoto et al. have reported ANA and RF positivity in 44% and 16%, respectively in their cohort of AIP patients [55]. Autoantibodies, such as, anti SS-A/Ro and SS-B/La or disease specific antimitochondrial antibodies are not commonly found [7]. Okazaki et al. [7] have attempted to provide theoretical support to the thesis that these self antigens may play a critical role in the pathogenesis of AIP. According to their scenario, an initial response to self-antigen (such as LF, CA-II, CA-IV, PSTI, amylase alpha, and PBP) is induced by decreased naïve T regulatory cells (or Tregs) and leads to the induction of a proinflammatory Th1 response and the release of IFN-*γ*, IL-1*β*, IL-2, and TNF-*α* [7]. Secondary to the initial Th1-predominant reaction, a Th2 response would lead the progression of the disease, which would include the production of IgG and IgG4, together with autoantibodies [7]. IL-10 and TGF-*β* may regulate IgG4 and fibrosis, and the classical complement pathway may be activated by the IgG1 immune complex [7]. A puzzling feature of the fine specificity of the autoantibodies found in patients in AIP is that they do not necessarily belong to the IgG4 subclass as at least those against PSTI mainly belong to the IgG1 subclass [50]. It is also puzzling as to why only the pancreas in involved in many cases, since many of the indicated autoantibodies are ubiquitous. As well, it would be expected that the prevalence of AIP would be much higher (especially as a concomitant condition with other autoimmune diseases) if the disease is characterized by a loss of tolerance to a variety of autoantigens. 

Zen et al. found that Th1 cells are predominant in the periphery of AIP patients, while Th2 cells were predominant in the affected organ [56]. That study also found that there was an overproduction of Th2 and increased CD4+CD25+FoxP3 Tregs in the organs of AIP patients [56]. In view of the fact that Tregs are involved in the production of IL-10, the hypothesis that type 1 AIP is characterized by an IL-10 mediated IgG4 class switching has been formulated [56]. As well, increased immune complexes are present in AIP, which is linked to increased IgG1 and low C3/C4, with a normal mannose-binding lectin [57]. These findings are in support of the hypothesis that the classical pathway of complement activation is involved in the pathogenesis of AIP [57]. 

Kawa et al. have tested their cohort of 44 AIP patients for the presence of autoantibodies and RF [58]. Thirteen out of 44 patients were RF positive. ANA at a titre of more than 1 : 40 were present in 54.5% (14/44) of the patients, 17 (38.6%) of them having ANA > 1 : 80 by IIF [58]. Anti-dsDNA antibodies were present in only 2/44 (4.5%) patients with AIP. SS-A and SS-B autoantibodies were totally absent [58]. Twenty one per cent of the patients had smooth muscle autoantibodies (SMAs) at a titre of more than 1 : 20, while only 2 had antimitochondrial antibodies [58]. Thyroglobulin and thyroid peroxidase autoantibodies were present in 7/41 (17.1%) and 5/41 (12.2%), respectively [58]. Overall, autoantibodies of any specificity were present in 79.5% (35/44) [58]. These data suggested that autoantibody markers are frequently present in patients with AIP, the most frequent autoantibody specificity being that against nuclear antigens. However, the target antigens of the ANA and SMA reactivities remain elusive, and SMA is not found in the majority of AIP cases. dsDNA may be a frequent target of autoantibody responses in autoimmune rheumatological diseases, but this appears unlikely in the case of AIP. The presence of a variety of autoantibody reactivities and several antigen specificities of the observed autoantibodies has led authors to speculate that the loss of tolerance seen in AIP is unlikely to be antigen driven. The investigation of the fine specificity of autoantibody reactivities in twin pairs, including individuals affected with AIP, may help us understand the origin of these responses and the immunopathogenesis of the disease. As well, twin studies may help elucidate to what degree environmental and genetic factors play a role in the disease development. Such studies have been useful in the understanding of other autoimmune conditions [59–61].

### 2.1. Antibodies to Carbonic Anhydrase and Lactoferrin

Anti-CA-IIAb and anti-LF antibodies are most frequently detected in AIP (54% and 73%, resp.) [45]. Aparisi et al. [47] investigated the role of CA-IIAb and IgG4 for the diagnosis of autoimmune pancreatitis. ELISA analysis for CA-IIAb followed by confirmatory western blot was performed in 227 subjects, comprised of 54 with idiopathic chronic pancreatitis (ICP), 54 age and sex-matched healthy controls, 86 with chronic alcoholic hepatitis and 33 with Sjögren's syndrome [47]. Increased serum CA-IIAb were present in 28% of ICP patients compared to 1.9% of healthy controls and 10.5% with chronic alcoholic pancreatitis [47]. Thus, the presence of CA-IIAb appears (at least in some extent) to relate to a state of pancreatic inflammation, irrespective of the stimuli responsible for the maintenance of pancreatic destruction. The finding of 64% of Sjögren's syndrome patients being seropositive for CA-IIAb clearly demonstrates that this autoantibody lacks disease specificity and cannot be used as a diagnostic marker for the confirmation of AIP in patients with a clinical suspicion of the disease [47]. When the analysis included the evaluation of IgG4, their levels were elevated in 15% ICP, 1.9% healthy controls, 8% chronic alcoholic pancreatitis, and 0% Sjögren's syndrome [47], suggesting that IgG4 is better at discriminating better AIP from Sjögren's syndrome with detectable CA-IIAb. Interestingly, all 4 ICP patients with increased CA-IIAb also had raised IgG4, cholestasis/jaundice at presentation, concomitant autoimmune disease, lymphoplasmacytic infiltration on histology, and prominent IgG4 infiltration [47]. Two of the 4 ICP patients had a positive response to corticosteroid therapy [47]. Similar results were obtained in another study which found increased CA-IIAb in 33% of ICP patients, in addition to those with Sjögren's syndrome [62]. Hardt et al. [63] found raised CA-IIAb and anti-LF in 22.9% ICP, which was also the case in 29.2% of type 1 diabetes mellitus (T1DM) patients. Other studies have also found raised CA-IIAb and/or anti-LF antibodies in AIP patients [45, 46]. At the experimental level in murine models of autoimmune sialadenitis and cholangitis, immunization with CA-II or LF induced the formation of systemic lesions (pancreatitis, sialadenitis, cholangitis, and interstitial nephritis) similar to IgG4-related disease [64, 65]. ANA and SMA have also been found in those studies, with 76% having ANA and 18% having SMA in one study [45], and 50% with ANA and 12% with SMA in another [46]. The relevance of these findings to the pathogenesis of AIP remains elusive in view of the lack of organ specificity of the observed reactivities. 

Investigation as to the role of other carbonic anhydrase isoenzymes, such as, CA-IV, IX, and XII (which are all normally expressed in pancreatic ductal cells) has been conducted in a study by Nishimori et al. [49]. Increased levels of CA-IV protein and peptide were found in patients with confirmed AIP (4/15 and 6/20, resp.), probable AIP (6/14 and 3/14), and Sjögren's syndrome (9/20 and 8/40) compared to none being detected in 26 healthy controls [49]. There was no difference noted between normal controls and pathological controls consisting of patients with chronic alcoholic pancreatitis and pancreatic cancer [49]. There was a significant correlation noted between the presence of serum antibodies to CA-IV and serum gamma-globulin and IgG levels in AIP patients [49]. There were no positive results in any groups in relation to CA-IX or CA-XII [49]. Although data suggests that antibodies to CA are present in patients with AIP, it should also be noted that these antibodies have also been found to a high degree in other conditions. If reactivity to CA is characteristic of AIP, it would be expected that other conditions with reactivity to CA (such as Sjögren's) would have a higher incidence of concomitant AIP and vice versa. This would also be the case for other autoantibody specificities being present in patients with AIP, such as, CA-IIAb. Whether these autoantibodies are just indicators of immune dysregulation and can be considered the end result of polyclonal activation characterizing autoimmune disorders. The association of AIP and autoimmune rheumatological conditions, such as, Sjögren's syndrome and SLE is rare, and this may reflect the lack of a significant presence of Sjögren's syndrome and SLE-related autoantibodies, like, the SS-A/Ro and SS-B/La and anti-dsDNA antibodies, respectively.

Significant homology between human CA-II and alpha CA of *Helicobacter pylori* (*H. pylori*) has been noted [66], and reactivity against a pancreatic homologue of Helicobacter has been demonstrated [54], suggesting that *H. pylori* infection may be involved in triggering AIP and AIP-related sclerosing cholangitis *via* mechanisms, such as, molecular mimicry, in individuals with a genetic predisposition [54, 66]. Molecular mimicry involving *H. pylori* and self antigens has been proposed to account for the immunopathogenesis of other cholestatic liver diseases, such as, primary biliary cirrhosis [67–69]. 

### 2.2. Antibodies to Amylase-2*α* and HSP-10: A Link with Type 1 Diabetes Mellitus?

Several studies have noted antibodies to amylase in AIP patients. Wiley and Pietropaolo [70] note that autoantibodies and autoreactive T cells in CD-28-deficient NOD mice (which develop AIP) recognized pancreatic amylase. Another study found that the administration of tolerogenic amylase-coupled fixed spleen cells reduced the severity of the disease [71]. Endo et al. have suggested that autoantibodies directed against amylase-2*α* may be a specific marker for AIP and T1DM. By ELISA, that group demonstrated that only AIP patients had reactivity to amylase-2*α*, compared to no reactivity in chronic alcoholic pancreatitis and pancreatic cancer [52]. Reactivity to amylase-2*α* was also observed in 88% T1DM, 21% acute-onset T1DM, and 6% T2DM [52]. Takizawa et al. [53] obtained 10 positive clones when the sera from AIP patients was screened through a human pancreas cDNA library. Seven of the 10 clones were amylase-2*α*, with 1 of the remaining 3 being identical to HSP-10 [53]. That same group developed an ELISA for detecting HSP-10 and found antibodies to HSP-10 in 92% of an AIP cohort and in 81% of patients with T1DM [53]. Antibodies to HSP-10 were present in only 8% of chronic alcoholic pancreatitis patients as controls and in 1.4% of healthy controls [53]. Larger studies are needed to establish the prevalence of antibodies against amylase-2*α*, as the pancreatic specificity of this antigen is of interest in the pathogenesis of AIP and T1DM. 

### 2.3. Antibodies to Plasminogen-Binding Protein

Frulloni et al. [54] screened a random peptide library with IgG from 20 patients with confirmed AIP. Peptide AIP1-7 was recognized by the serum of 18 out of 20 (90%) AIP patients, 4 of 40 (10%) patients with pancreatic cancer, and in none of the healthy controls. This peptide demonstrated homology with the PBP of *H. pylori* and with ubiquitin-protein ligase E3 component n-recognin 2, which is highly expressed in the pancreatic acinar cells [54]. Antibodies against PBP were present in 95% of AIP patients, as well as in 10% of patients with pancreatic cancer [54]. No antibodies were present in patients with chronic alcoholic pancreatitis or those with intraductal papillary mucinous neoplasm [54]. A second series had similar results with 93% of AIP patients and 1% of patients with pancreatic cancer having antibodies to the PBP peptide. Original antibody testing was performed using DELFIA, a time-resolved fluorescent assay. One of the limitations of the study was that the concentration used was relatively high (20 *μ*g), and the sera were tested in 1 in 50 dilution, raising the possibility that the observed reactions were due to hyperglobulinaemia characterizing AIP. However, this appeared unlikely, as the authors clearly demonstrated that sera from patients with autoimmune-rheumatic diseases were totally unreactive. Subsequent experiments were based on ELISA and western blotting and confirmed the presence of anti-PBP antibodies. These findings have led Frulloni et al. to suggest that pancreatic acinar cells may be the target of autoimmune attack in AIP, but that antibodies to PBP could not be used to differentiate AIP from pancreatic cancer [54]. Anti-PBP antibodies warrant further investigation, as cross reactivity with ubiquitin-protein ligase E3 component n-recognin 2 may account for pancreatic specificity. These findings must be interpreted with caution, as the prevalence of anti-PBP antibodies has not been investigated in great detail. In fact, larger multicenter studies are needed to confirm the significance of the diagnostic and clinical relevance of anti-PBP antibodies in AIP patients. 

### 2.4. Anti-Pancreatic Secretory Trypsin Inhibitor

Asada et al. [50] have considered PSTI as a potential target autoantigen in AIP. They based their hypothesis on data suggesting that endogenous trypsin inhibitor and mutations in PSTI are closely associated with the pathogenesis of hereditary pancreatitis and idiopathic chronic pancreatitis [55]. These Investigators [50] noted that 42.3% of AIP patients had antibodies to PSTI by immunoblotting and 30.8% by ELISA, compared to none of the controls. Both assays were developed *in house* for the purpose of this study. The serum dilution used for the ELISA testing was 1 : 40, a dilution which is generally considered inadequate for proper antibody detection. Also, the mean absorbance values of the ELISA testing were relatively low (0.27 ± 0.19). However, the authors were able to demonstrate the presence of anti-PSTI antibodies by immunoblotting in 1 : 1000, indicating that AIP patients react with PSTI [50]. The same group of researchers investigated the immune responses of mice injected with polyinosinic polycytidylic acid, which accelerates the development of pancreatitis [72]. The severity of the pancreatitis in the mice was graded histologically, followed by immunohistological examination and analysis of serum autoantibodies by ELISA [72]. Histologically, there was a rich infiltration of B cells and CD138 plasmacytes in the pancreatic tissue [72]. A variety of autoantibodies were present in these mice, including anti-PSTI (91.7% of mice) [72]. This finding is intriguing based on the above-mentioned data, as it is indicative of PSTI being an autoantigen in an animal model of the disease, as well as in humans. In fact, anti-PSTI were more prevalent than anti-CA-IIAb (33.3%) and anti-LF (45.8%) [72]. No antibodies were found against glutamic acid decarboxylase, suggesting that the loss of tolerance seen in AIP is antigen driven [72]. The epitope of the anti-PSTI antibodies was determined to be the site of PSTI which is involved in the suppression of trypsin activity [72]. This is of interest as the group led by Löhr (see above) found that the serum of AIP patients contained high titers of autoantibodies against trypsinogens, including those to PSTI [51]. Further investigation is needed to identify the prevalence of anti-PSTI antibodies in AIP patients. The same Investigators tested serum samples for various other autoantibodies. Among their 26 patients with AIP, 19 (73.1%) had anti-LF antibodies and 18 (69.2%) had ANA. Anti-CA-IIAb and RF was found in 14 (53.8%) and 6 (23.1%), respectively, while SMA were present in only 4 (15.4%) of the AIP patients. Asada et al. [50] have also tested for reactivity to insulin-dependent diabetes and found that just 1 (3.8%) of the patients had antibodies against antiglutamic acid decarboxylase and anti-islet cell antibody each. None of the patients had detectable AMA. They concluded that the diagnostic sensitivity increases from 73.1 to 76.9% by a combination of anti-LF and anti-PSTI antibodies. No data have been provided for the specificity of these autoantibodies as the study did not include tests in pathological and healthy controls, and this is one of its limitations. 

Further support for the organ-specific autoimmune attack in AIP has been presented by Löhr et al. [51], which examined the expression of proteins involved in the inflammatory process in a murine AIP model, as well as in the pancreatic tissue of 12 AIP patients and 8 patients with non-AIP chronic pancreatitis. That group identified 272 upregulated genes involved with immunoglobulin, chemokine and chemokine receptor production [51]. As well, 86 genes encoding pancreatic proteases were downregulated, and trypsin-positive acinar cells were virtually absent [51]. The sera of AIP patients contained high titers of antibodies against the trypsin inhibitor PST1, and similar results were found in the murine AIP model [51]. The same study has found elevated titers of autoantibodies against trypsinogens PRSS1 and PRSS2 but not against PRSS3. ELISA testing based on recombinant antigens revealed significantly increased levels at 1/600 serum dilution, particularly against trypsinogen PRSS1 in AIP patients compared to non-AIP chronic pancreatitis patients or normal controls. These results have led the authors to suggest that a loss of tolerance to and production of antibodies against trypsinogens (and likely other pancreatic antigens) are probably involved in the pathogenesis of AIP and may therefore provide useful diagnostic targets. Their data clearly support the notion that the autoimmune attack in AIP is not only directed to the ductal cell constituents but also against the acinar cell components, such as, the trypsinogens (PRSS1 and PRSS2) and PSTI, but these data require external validation. 

## 3. Conclusion

A variety of autoantibodies have been found in the sera of patients with AIP. Presently, none of these autoantibodies appear to be disease specific. It is possible that a loss of tolerance to a variety of pancreatic specific and nonpancreatic-specific antigens may be involved in the initiation of AIP. However, this does not explain the pancreatic specificity of the disease, as they may also be present in other conditions, such as, Sjögren's syndrome, as well as in pancreatic cancer. It would be expected that a loss of tolerance to an ubiquitously expressed antigen would result in a higher prevalence of AIP in conditions that are characterized by specific autoantibodies, which is not the case. With that said, the extrapancreatic lesions encountered in AIP may be indicative of loss of tolerance to an ubiquitous antigen. Organ-specific immunological targets in AIP may include PSTI, amylase-2*α*, or other currently unknown pancreatic antigens. Further studies are needed to clarify whether loss of tolerance to these antigens plays a role in the immunopathogenesis of AIP. Additionally, molecular mimicry with *H. pylori* antigens and possibly other microbial antigens may also be involved in the loss of tolerance, especially in regards to PBP which shares homologous regions with pancreatic ubiquitin-protein ligase E3 component n-recognin 2. Further investigation is needed to characterize autoantibodies present in AIP, in addition to their clinical and diagnostic significance. 

## Figures and Tables

**Table 1:** Main disease-related autoantibody specificities in autoimmune pancreatitis. Several autoantibodies have been detected in the sera of patients with autoimmune pancreatitis (AIP). Although none have been established to be disease specific, it appears that a loss of tolerance to several pancreatic antigens may be involved in the initiation of AIP. The prevalence of antibodies against carbonic anhydrase, lactoferrin, heat-shock protein 10, amylase alpha, plasminogen-binding protein, and pancreatic secretory trypsin inhibitor antigens, key references of studies investigating autoantibodies against these antigens as well as the origin of the cohorts are provided. Information regarding conventional autoantibodies, such as, antinuclear and smooth muscle autoantibodies is provided within the text.

Autoantibody/antigen	Number of AIP sera tested	Patient origin	Frequency in confirmed AIP (%)	Frequency in controls	Reference
Anticarbonic Anhydrase-II	17	Japanese	59	—	[45]
	54	Japanese	28	1.9 healthy controls, 10.5 chronic alcoholic pancreatitis, 64 Sjogren's	[47]
	33	Japanese	33	62 Sjögren's	[62]
	48	European	12.5	0 healthy controls	[63]
Antilactoferrin	17	Japanese	76	—	[45]
	48	European	20.8	0	[63]
Anticarbonic Anhydrase-IV	—	Japanese	27 (protein), 30 (peptide)	0 healthy controls, 45 (protein) Sjögren's, 20 (peptide) Sjögren's	[49]
Heat-shock protein 10	—	Japanese	92	81 type 1 diabetes mellitus, 8 chronic alcoholic pancreatitis, 1.4 healthy controls	[53]
Amylase-2*α*	15	Japanese	100	88 type 1 diabetes mellitus, 6 type 2 diabetes mellitus, 0 chronic alcoholic pancreatitis and pancreatic cancer	[52]
Plasminogen-binding protein antibodies	20	European	95 (93 in second series)	10 pancreatic cancer (1 in second series), 0 chronic alcoholic pancreatitis and intraductal papillary mucinous neoplasm	[54]
Antitrypsinogens	19	German	79 on ELISA	10 non-AIP chronic cholangitis and healthy controls	[51]
Antipancreatic secretory trypsin inhibitor	26	Japanese	42.3 on western blot, 30.8 on ELISA	0	[50]

## Abbreviations

AIP: Autoimmune pancreatitis
ANA: Antinuclear antibody
anti-PBP: Antiplasminogen-binding protein peptide
anti-PSTI: Antipancreas secretory trypsin inhibitor
CA: Carbonic anhydrase
*H. Pylori*: *Helicobacter pylori*
HSP-10: Heat-shock protein 10
ICP: Idiopathic chronic pancreatitis
LF: lactoferrin;
T1DM: Type 1 diabetes mellitus
Th: T-helper
TGF-*β*: Transforming growth factorbeta
Treg: Regulatory T-cells.
